# Are Extracted Materials Truly Representative of Original Samples? Impact of C18 Extraction on CDOM Optical and Chemical Properties

**DOI:** 10.3389/fchem.2016.00004

**Published:** 2016-02-05

**Authors:** Andrea A. Andrew, Rossana Del Vecchio, Yi Zhang, Ajit Subramaniam, Neil V. Blough

**Affiliations:** ^1^Department of Chemistry and Biochemistry, University of MarylandCollege Park, MD, USA; ^2^Earth System Science Interdisciplinary Center, University of MarylandCollege Park, MD, USA; ^3^Lamont Doherty Earth Observatory at Columbia UniversityPalisades, NY, USA

**Keywords:** CDOM absorbance, fluorescence, C18 extraction, extraction efficiency, NaBH_4_ reduction

## Abstract

Some properties of dissolved organic matter (DOM) and chromophoric dissolved organic matter (CDOM) can be easily measured directly on whole waters, while others require sample concentration and removal of natural salts. To increase CDOM content and eliminate salts, solid phase extraction (SPE) is often employed. Biases following extraction and elution are inevitable, thus raising the question of how truly representative the extracted material is of the original. In this context, we investigated the wavelength dependence of extraction efficiency for C18 cartridges with respect to CDOM optical properties using samples obtained from the Middle Atlantic Bight (MAB) and the Equatorial Atlantic Ocean (EAO). Further, we compared the optical changes of C18 extracts and the corresponding whole water following chemical reduction with sodium borohydride (NaBH_4_). C18 cartridges preferentially extracted long-wavelength absorbing/emitting material for samples impacted by riverine input. Extraction efficiency overall decreased with offshore distance away from riverine input. Spectral slopes of C18-OM samples were also almost always lower than those of their corresponding CDOM samples supporting the preferential extraction of higher molecular weight absorbing material. The wavelength dependence of the optical properties (absorption, fluorescence emission, and quantum yield) of the original water samples and their corresponding extracted material were very similar. C18 extracts and corresponding water samples further exhibited comparable optical changes following NaBH_4_ reduction, thus suggesting a similarity in nature (structure) of the optically active extracted material, independent of geographical locale. Altogether, these data suggested a strong similarity between C18 extracts and corresponding whole waters, thus indicating that extracts are representative of the CDOM content of original waters.

## Introduction

The optical properties of chromophoric dissolved organic matter (CDOM; and humic substances, HS) have been intensively studied over the last several decades. However, inter-laboratory data comparison is often impossible due to various methods employed to collect, extract, and analyze DOM and CDOM. Despite a few very sensitive analytical techniques such as optical spectroscopy which allow direct measures of bulk CDOM in natural waters, the majority of the available techniques require prior extraction, concentration of DOM/CDOM, and salt removal.

Many solid phase extraction (SPE) techniques for DOM isolation from fresh to salt waters have thus been widely used including cartridges pre-packed with XAD resins (Daignault et al., 1988), C18-bonded silica sorbents (Benner, 2002 and references therein), and more recently, modified styrene-divinylbenzene copolymer type sorbents (PPL) (Dittmar et al., 2008). Reverse osmosis (RO) has also been used for retention and concentration of DOM from fresh waters (Serkiz and Perdue, 1990). Most recently, a new technique for more efficient isolation of DOM from seawater was developed by combining RO with electrodialysis (RO/ED; Vetter et al., 2007). However, extraction efficiencies vary among these techniques and are routinely expressed relative to dissolved organic carbon (DOC).

XAD resins have been widely used to remove hydrophobic compounds of various molecular sizes. International Humic Substances Society (IHSS) has used XAD-8 to extract standard reference materials (Suwannee River Fulvic and Humic Acids, SRFA, and SRHA) from natural waters. However, XAD-8 resins used for the extraction of DOM are either no longer available (Dittmar et al., 2008) or are difficult to obtain and require extensive purifications before use (IHSS). C18 sorbents have been more widely employed for reversed phase extraction of nonpolar to moderately polar compounds from water. Retention is based on the partitioning of the nonpolar organic analyte into the nonpolar sorbent. Thus, charged compounds are usually not retained and require ion exchange SPE for their extraction. Sequential SPE through two different XAD resins has been shown to recover comparable DOC as silica-C18, with percent DOC recovery ranging from 23 to 40% (Amador et al., 1990; Druffel et al., 1992). More recently, PPL sorbents have been employed for extracting DOM. As with XAD and silica-C18, PPL retains moderately polar to nonpolar substances from large volumes of water. Dittmar et al. (2008) showed that the use of PPL was more efficient in the extraction process, reporting that an average 62% of DOC was recovered as salt-free extracts. The most recent extraction methods using RO/ED has been shown to have DOC recoveries of 60% (Vetter et al. (2007). Similarly, Helms et al. (2013) reported DOC recoveries of 78 ± 3% and fluorescence recoveries in the EEM Peak C and M regions roughly in proportion to DOC, for 674 m Pacific waters extracted by RO/ED.

These extraction processes facilitate the elimination of the high concentrations of inorganic salts occurring in marine waters. Thus, highly concentrated organic samples with relatively low salt content can be generated and different analytical techniques can be performed on the extracts. However, most of the studies have mainly focused on recovery/extraction efficiency in terms of % DOC. Extraction efficiency of carbon (% DOC) would not necessarily match extraction efficiency of CDOM, the carbon pool that absorbs light, given the variable dependence of CDOM to DOC. Further, DOC may be contaminated with carbon from solvents employed for elution. On this basis, the question as to how representative the extracts reflect the properties of the original samples as a whole needs further consideration.

Some earlier studies have made attempts to better address this question by comparing the optical properties of whole natural water collected from different environments to their corresponding C18 extracts (C18-OM). Green and Blough (1994) employed water samples from southern and western coast of Florida to the eastern Gulf of Mexico, and compared spectral slopes, absorption coefficient at 355 nm and fluorescence quantum yields for CDOM and C18-OM. They also examined the efficiency of extraction of the various water types. Their results indicated that longer wavelength absorbing material was isolated with greater efficiency, which was reflected in lower S values for C18 extracts relative to CDOM. Similarly, fluorescence results also indicated greater retention of longer wavelength fluorescing components, 65% of that excited at 450 nm, compared to 35% at 325 nm. Boyle et al. (2009) investigated the optical properties of CDOM and C18-OM, using samples from the Middle Atlantic Bight (MAB), but only compared a_CDOM_(355) and spectral slopes. Helms et al. (2013) reported minor effects of RO/ED on the optical properties of SUVA254, spectral slope, and fluorescence indices for extracts of Pacific waters. Their results showed a slight increase in S275-295 slope ratio and decrease in Peak A and M fluorescence. These studies (Green and Blough, 1994; Boyle et al., 2009; Helms et al., 2013) were limited in terms of (1) the number and types of samples studied; (2) limited number wavelengths examined; and (3) did not explore the effect, if any, of the preferential extraction of long wavelength absorbing and fluorescing components on the chemical properties and reactivity of C18 extracts relative to CDOM.

In this study, we present a more extensive comparison of the optical properties of original CDOM and C18-OM samples. This study further examined the changes in the optical properties of CDOM and C18-OM following chemical reduction with sodium borohydride (NaBH_4_). Samples were collected from very different geographic locales spanning riverine, estuarine, and marine type environments: (a) along a transect from the Delaware River to the Sargasso Sea (Middle Atlantic Bight, MAB); and (b) across the Equatorial Atlantic Ocean (EAO) from the open ocean gyre to the Congo River Plume. To our knowledge no study has done such a detailed comparison from such a wide range of water types. Absorption and fluorescence were measured directly for natural waters (CDOM) and compared to those of C18 extracts (C18-OM); similarly, absorption and fluorescence were measured directly for NaBH_4_ reduced CDOM and compared to NaBH_4_ reduced C18-OM. The wavelength dependence of extraction efficiency of C18 cartridges was examined in relation to the optical properties (MAB and EAO).

## Materials and procedures

### Samples

**Figure 1:**
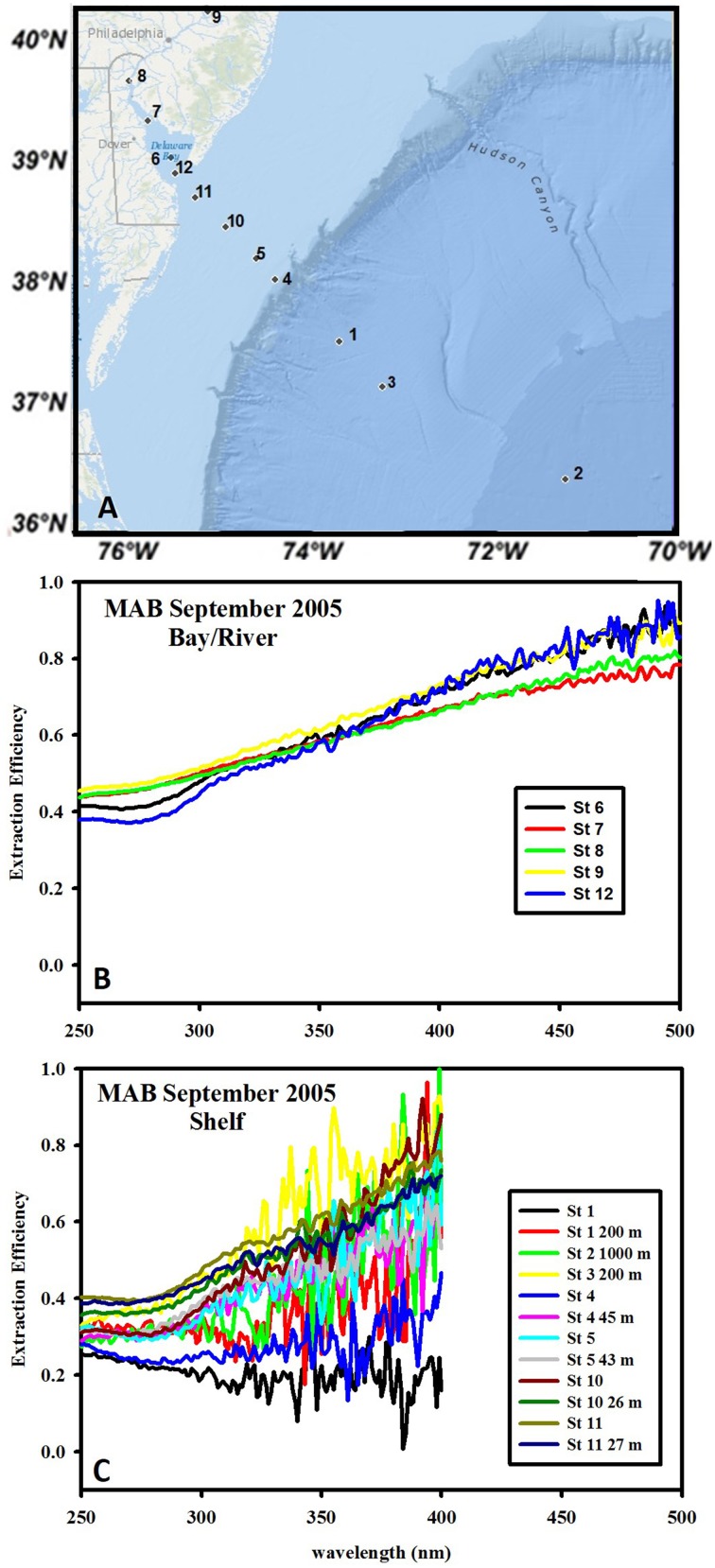
**Wavelength dependence of extraction efficiency of C18 cartridges for waters from the MAB during September 2005**. **(A)** Typical transect for all cruises, extending from the Delaware River to shelf/offshore waters. **(B)** Extraction efficiency for river and bay samples. **(C)** Extraction efficiency for shelf and shelf break samples. The large noise at longer wavelengths is due to the small absorbance values. Values at >~400 nm are at or below the detection limit and were thus omitted.

Samples from the Mid Atlantic Bight (MAB) were collected onboard the R/V Cape Henlopen and R/V Cape Hugh Sharp from August to December during three cruises from 2005 to 2006: September 20–24, 2005; August 24–28, 2006; and November 30–December 4, 2006. A typical transect from the Delaware River (~40 N; −75 W) to the western boundary of the Gulf Stream (~36 N; −72 W) was visited during all cruises (Figure 1A). The water column was thermally- and density-stratified during the summertime, typical of coastal waters on the shelf of the MAB, while stratification was absent or not fully developed during the spring, fall, and winter surveys (Del Vecchio and Blough, 2004b). Ocean samples were collected onboard the R/V Endeavor in the EAO in May-June 2009 over a 5 week period, encompassing three zonal and meridional sections between 23 W and 5 E, and 3 N and 3 S, respectively (Andrew et al., 2013).

Water samples for the optical measurements of CDOM were collected as previously reported (Del Vecchio and Blough, 2004b). Briefly, samples were collected employing a CTD (conductivity–temperature–depth) rosette equipped with Niskin bottles and were immediately filtered using GF/F filters (0.7-μm pore size). Samples for optical measurements were stored in the dark at 4°C until measurement (about 2 weeks for coastal samples and 3 months for ocean samples). Before measurement, samples were re-filtered through 0.2-μm pore-size nylon syringe filters to ensure removal of all particles.

Water samples for optical measurements of C18 extracted material were collected from the vessel's surface water pumping system (2 and 5 m) and CTD rosette and were immediately filtered through an in-line Gelman fluted capsule filter (0.2 μm pore size). A clean Teflon line for the pumping system was supplied at the beginning of each cruise to prevent contamination by material accumulating in the line. Filtered water samples (20 L) were acidified to pH 2 (pre extraction) and then pumped through the SPE cartridge (C18 extraction column, UCT) at the flow rate of 50 mL min^−1^ as described by Boyle et al. (2009). The cartridges were pretreated with 100 ml of high purity methanol followed by 50 mL of acidified (pH 2) Milli-Q water prior to extraction. After extraction each cartridge was rinsed with 1 L of acidified (pH 2) Milli-Q water to remove salts and stored in the refrigerator (4°C) until further processing. The post cartridge eluent was stored for further analysis (post extraction).

DOM was extracted from the C18 cartridges with 50 mL of high purity methanol: the first fraction (5 mL) that contained some aqueous residue was separated from the second fraction (45 mL). The second fraction was collected into a 100 mL round bottom flask and evaporated to dryness under vacuum using a rotary evaporator at 30–35°C. The dried material was re-dissolved with Milli-Q water (~2 mL), neutralized with diluted NaOH solution to pH 7 and stored frozen till further analysis. This material is here on referred to as C18-OM.

### Optical measurements

CDOM absorption spectra were acquired with a Shimadzu 2401-PC spectrophotometer employing a 10 cm optical cell (1 cm cell for C18-OM) using Milli-Q water as the blank, as reported previously (Vodacek et al., 1997; Del Vecchio and Blough, 2004b). Absorption spectra for offshore CDOM samples (including samples from the EAO as well as samples further offshore in the MAB region) were obtained on a liquid core capillary waveguide long path spectrometer (WPI) using the 10, 50, or 200 cm fiber optic cell. Salinity-matched solutions were used as reference to avoid absorbance baseline offsets caused by refractive index differences between reference and sample. Absorption spectra were recorded over the range 200–800 nm. Absorption coefficients *a*(λ) were calculated using the following equation,

(1) $$\begin{array}{l} a\left(\lambda \right)=2.303A\left(\lambda \right)∕L \end{array}$$

as in Blough and Del Vecchio (2002), where *A*(λ) is the absorbance over pathlength, *L*. The spectra were then fit to an exponential function,

(2) $$\begin{array}{l} a\left(\lambda \right)=a\left(\lambda_{0}\right)e^{-S\left(\lambda -\lambda_{0}\right)} \end{array}$$

using a nonlinear least squares fitting routine (NLF) over the range 300–700 nm (Blough and Del Vecchio, 2002). Here λ_0_ is a reference wavelength and *S* is the spectral slope parameter.

Optical measurements for C18-OM samples were collected for all EAO samples, and for 23 of the 43 C18-OM samples for the MAB (as they were the only ones available in storage). However, these samples more than adequately represented all the various water types under study.

The wavelength dependence of C18 extraction efficiency was calculated using Equation (3) below, where pre extraction acidified waters refer to the pH 2 water before passage through the C18 column, while post extraction acidified waters refer to the pH 2 water collected after passage over the column.

(3) $$\begin{array}{l} Extraction\;efficiency\\ =\;1\;-\;\frac{Abs\;\left(post\;extraction\;acidified\;waters,\;pH\;2\right)}{Abs\;\left(pre\;extraction\;acidified\;waters,\;pH\;2\right)} \end{array}$$

The wavelength dependence of extraction efficiency was only obtained for the MAB samples, because the post extraction waters for EAO samples were not collected at the time of extraction.

CDOM and C18-OM fluorescence measurements were acquired with an Aminco–Bowman AB2 luminescence spectrometer employing a 1-cm optical cell and Milli- Q water as blank. C18-OM samples were diluted prior to acquiring fluorescence measurements in order to avoid inner filter effects, while CDOM samples were measured as is. Both the excitation and emission monochromator bandpasses were set to 4 nm for extracts (C18-OM) and 8 nm for the natural waters (CDOM). The emission spectra were recorded from 10 nm greater than λ_exc_ to 700 nm, with λ_exc_ incremented every 10 nm over the range 290–600 nm. The spectra were corrected for the instrument response using factors supplied by the manufacturer. Fluorescence emission was normalized to the integrated emission of 1 ppb quinine sulfate with excitation at 350 nm and reported in quinine sulfate equivalents (QSE). The wavelength dependence of fluorescence emission maxima (λ_max_) and apparent quantum yields (ϕ) were measured as described in Del Vecchio and Blough (2004a) and (Green and Blough, 1994), respectively. Fluorescence quantum yields were calculated relative to quinine sulfate (QS) as a reference (λ_exc_ 350 nm) in 0.1 N H_2_SO_4_:

(4) $$\begin{array}{l} \phi \left(\lambda \right)=\frac{F^{\prime }\left(\lambda \right).{A\left(\lambda \right)}_{r}}{F^{\prime }{\left(\lambda \right)}_{r}.A\left(\lambda \right)}\cdot 0.54 \end{array}$$

Subscript *r* refers to the reference (Quinine sulfate), *A*(λ) is the absorbance at the excitation wavelength, and *F*′ is the integrated corrected fluorescence emission produced by excitation at wavelength λ and 0.54 is the QS quantum yield (Green and Blough, 1994).

### NaBH_4_ reduction

Selected CDOM and corresponding C18-OM samples were chosen for the NaBH_4_ reduction analysis based on geographic locale. These samples consisted of waters from river (0 ppt), bay (17.6 ppt), shelf (32.5 ppt), and open ocean (35.4 ppt) regions.

Reduction with NaBH_4_ (Fisher) was performed as described by Andrew et al. (2013). Briefly, natural waters (20.0–30.0 mL) were placed in a glass vial, capped, and sparged with ultrapure N_2_ for 35 min. Solid NaBH_4_ was added to the vial using a one-to-one ratio of NaBH_4_ (mg) to sample volume (mL) (resulting in very large mass excess of NaBH_4_ relative to CDOM to compensate for the large ratio of water to organic matter in these samples). NaBH_4_ was added under continuous N_2_ flow. C18-OM was reduced in a 1 cm quartz cuvette using 3.0 mL of sample and mass excess of NaBH_4_ (~50 fold) based on approximate organic matter content (derived from matching absorbance spectra to that of known concentrations of SRFA). This was accomplished by adding ~85–95 μL of a concentrated NaBH_4_ stock solution (~45.7 mg NaBH_4_ in 3.0 mL of Milli-Q at pH 12) to the C18-OM sample. The reduction was considered to be complete when no further changes in absorption spectra were observed (~24 h). Sample pH increased from pH 7–8 to pH 10–11 due to the reaction of excess NaBH_4_ with water (Tinnacher and Honeyman, 2007; Ma et al., 2010; Golanoski et al., 2012). Thus, sample pH was readjusted to the original pH to allow for comparison with the untreated natural water sample.

Optical properties were measured prior to and following reduction and reported relative to the untreated sample as *A*_*f*_ (fraction of absorbance remaining), Δ*F* (change in fluorescence emission), and *F*_*f*_ (fraction of fluorescence emission remaining), defined by the following equations:

(5) $$\begin{array}{rcl} A_{f} & = & \frac{A\left(t\right)}{A\left(0\right)} \end{array}$$

(6) $$\begin{array}{rcl} △F & = & F\left(t\right)-F\left(0\right) \end{array}$$

(7) $$\begin{array}{rcl} F_{f} & = & \frac{F\left(t\right)}{F\left(0\right)} \end{array}$$

where *A*(*t*) is the absorbance of the treated sample and *A*(0) is the absorbance of the original sample. Likewise, *F*(*t*) and *F*(0) are the fluorescence emission spectrum of the treated and untreated sample, respectively.

## Results and discussion

### Extraction efficiency

The C18 extraction efficiency was calculated for MAB waters only (Figure 1) as post extraction waters for EAO samples were not stored at the time of collection. The C18 extraction efficiency for inshore MAB waters (river and bay) was approximately 0.4–0.45 at 250 nm and monotonically increased with increasing wavelengths to ~0.9–1.0 at 500 nm, thus clearly exhibiting a preferential retention of the long-wavelength absorbing material from the bulk CDOM (Figure 1B) consistent with previous work (Green and Blough, 1994). Offshore samples clearly exhibited lower extraction efficiency (~0.25–0.3) at short wavelength < 300 nm that sometimes, but not always, increased with increasing wavelengths ranging from 0.3 to 0.8. The varying wavelength dependence of the extraction efficiency for offshore samples at longer wavelengths was possibly impacted by the occasionally extremely low CDOM absorption values for these samples (at or below the detection limit) resulting in higher errors in the calculated values for extraction efficiency (Figure 1C). Overall the extraction efficiency was higher for fresh (river) waters than for offshore waters at short wavelengths and increased with increasing wavelength for fresh (river, bay) waters while this trend was more variable for offshore waters.

### Absorbance and spectral slope

**Figure 2:**
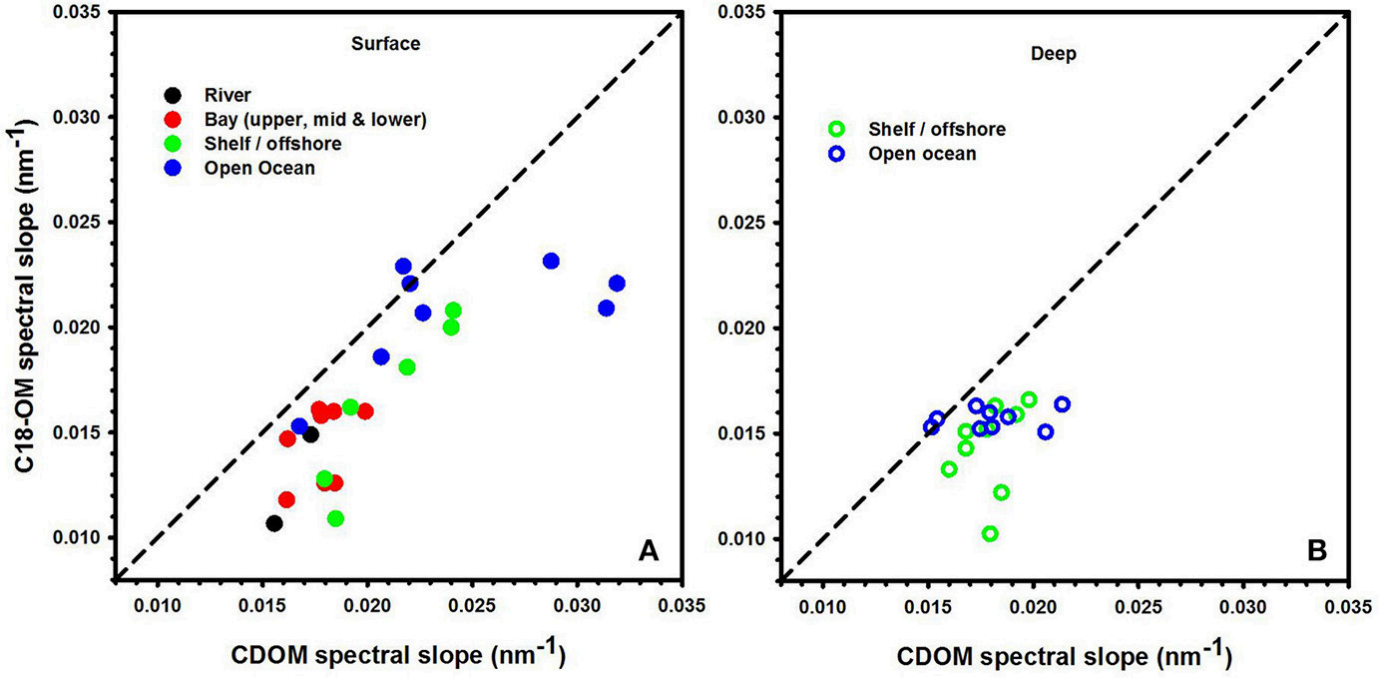
**Spectral slope values acquired for CDOM and C18-OM for river, bay, shelf, and open ocean waters from the MAB and EAO**. Solid symbols represent surface samples **(A)**. Open symbols represent depths below the surface **(B)**. 1:1 line is also shown.

The spectral slopes for C18-OM samples were lower relative to those for CDOM samples (Table 1 and Figure 2) indicating enrichment of long wavelength absorbing components by the extraction process. It was also generally observed that spectral slope values for surface CDOM samples increased from river to open ocean, ranging from 0.015 to ~0.030 m^−1^, respectively (Figure 2A). Contrarily, spectral slope values for CDOM samples below the surface remained within a much narrower range of 0.016–0.020 m^−1^ (Figure 2B). A similar trend of increasing slopes from river to ocean surface samples was generally observed for C18-OM though the overall range was shifted toward slightly lower values. This observation supports the finding that lower molecular weight material appears to be less efficiently extracted by C18 cartridges. In addition, the C18-OM spectral slopes were closer to the CDOM spectral slope values for most of the surface ocean samples (Figure 2A, closer to the one-to-one line) relative to shelf and river waters, possibly indicative of a more uniform extraction in offshore waters. However, a few surface ocean samples did not follow this trend, which may have resulted from poorer non linear fits as a result of low absorbance in surface ocean waters.

**Table 1:** **Spectral slope values (S, nm^−1^) for MAB samples in August, December, and September 2006, and EAO samples in June 2009**.

**Sample**	**Geographic locale**	**Position**		**Salinity (ppt)**	**S (nm**^**−1**^**)**			**Sample**	**Position**		**Salinity**	**S (nm**^**−1**^**)**	
		**Lat**.	**Long**.						**Lat**.	**Long**.			
**MAB August**					**CDOM**	**C18-OM**		**EAO (5 m)**				**CDOM**	**C18-OM**
St 1 2 m	Bay mouth	38.9	−75.1	29.6	0.0184	0.0160		St 22	3.0	−23.0	34.6	NA	NA
St 1 17 m	Bay mouth	38.9	−75.1	31.5	0.0178	0.0152		St 26	3.50	−10.00	34.4	0.0217	0.0229
St 4 2 m	Shelf	38.0	−74.0	31.6	0.0219	0.0181		St 48	−5.00	−10.00	35.3	0.0227	0.0207
St 4 75 m	Shelf	38.0	−74.0	35.1	0.0168	0.0143		St 51	−5.00	0.00	35.2	0.0319	0.0221
St 7 1000 m	Gulf stream	36.2	−71.8	35.1	0.0160	0.0133		St 61	−0.67	0.00	35.4	0.0314	0.0209
St 10 2 m	Mid shelf	38.2	−74.3	31.5	0.0240	0.0200		St 73	3.00	0.00	34.7	0.0288	0.0232
St 10 35 m	Mid shelf	38.2	−74.3	33.1	0.0168	0.0151		St 75	3.00	5.00	35.1	0.0220	0.0221
St 12 2 m	Lower bay	39.0	−75.1	28.4	0.0199	0.0160		St 89	−2.00	5.00	36.1	0.0207	0.0186
St 14 2 m	Mid bay	39.3	−75.4	17.6	0.0177	0.0161		St 96	−6.00	5.00	34.1	0.0168	0.0153
St 19 2 m	River	40.1	−74.8	0.1	0.0173	0.0149							
St 20 2 m	Mid bay	39.3	−75.4	12.5	0.0177	0.0166		**EAO (1000m)**	**Lat**.	**Long**.	**Salinity**	**CDOM**	**C18-OM**
								St 22	3.0	−23.0	34.6	0.0179	0.0160
**MAB December**		**Lat**.	**Long**.	**Salinity**	**CDOM**	**C18-OM**		St 26	3.5	−10.0	34.7	0.0188	0.0158
St 2 2 m	Offshore	38.00	−74.05	33.7	0.0192	0.0162		St 48	−5.0	−10.0	34.6	0.0214	0.0164
St 2 50 m	Offshore	38.00	−74.05	33.8	0.0192	0.0159		St 51	−5.0	0.0	34.6	0.0175	0.0152
St 3 30m	Shelf	38.18	−74.25	33.7	0.0192	0.0130		St 61	−0.7	0.0	34.6	0.0206	0.0151
St 4 20 m	Bay mouth	38.68	−74.89	31.8	0.0181	0.0104		St 73	3.0	0.0	34.6	0.0152	0.0153
St 6	Bay mouth	38.88	−75.09	26.44	0.0180	0.0126		St 75	3.0	5.0	34.6	0.0180	0.0153
St 6 15m	Bay mouth	38.88	−75.09	28.3	0.0180	0.0102		St 89	−2.0	5.0	34.6	0.0173	0.0163
St 7	River	39.97	−75.13	0.08	0.0156	0.0107		St 96	−6.0	5.0	34.6	0.0154	0.0157
St 8	Upper bay	39.58	−75.55	0.09	0.0162	0.0147							
St 9	Shelf	38.44	−74.57	32.53	0.0185	0.0109		**MAB September**	**Lat**.	**Long**.	**Salinity**	**CDOM**	**C18-OM**
St 9 20 m	Shelf	38.44	−74.57	32.5	0.0185	0.0122		St 2 1000m	36.5	−71.2	36.0	0.0198	0.0166
St 11	Lower bay	38.99	−75.14	23.28	0.0180	0.0128		St 5	38.2	−74.3	32.5	0.0241	0.0208
St 13	Upper bay	39.62	−75.58	0.12	0.0162	0.0118		St 5 41m	38.2	−74.3	32.5	0.0182	0.0163
St 14	Bay mouth	38.88	−75.09	28.66	0.0185	0.0126		St 7	39.3	−75.4	14.4	0.0178	0.0158

*NA, data not available*.

Consistent with previous work, S has been shown to decrease with increasing molecular size (Green and Blough, 1994; Boyle et al., 2009; Yan et al., 2012; Sharpless and Blough, 2014). Thus, lower values of S for C18-OM is suggestive of the presence of higher molecular weight material, implying the preferential extraction/elution of larger size material. This is a reasonable expectation, as larger material is generally considered to be more nonpolar, more easily adsorbed and thus extracted.

### Fluorescence emission and quantum yield

**Figure 3:**
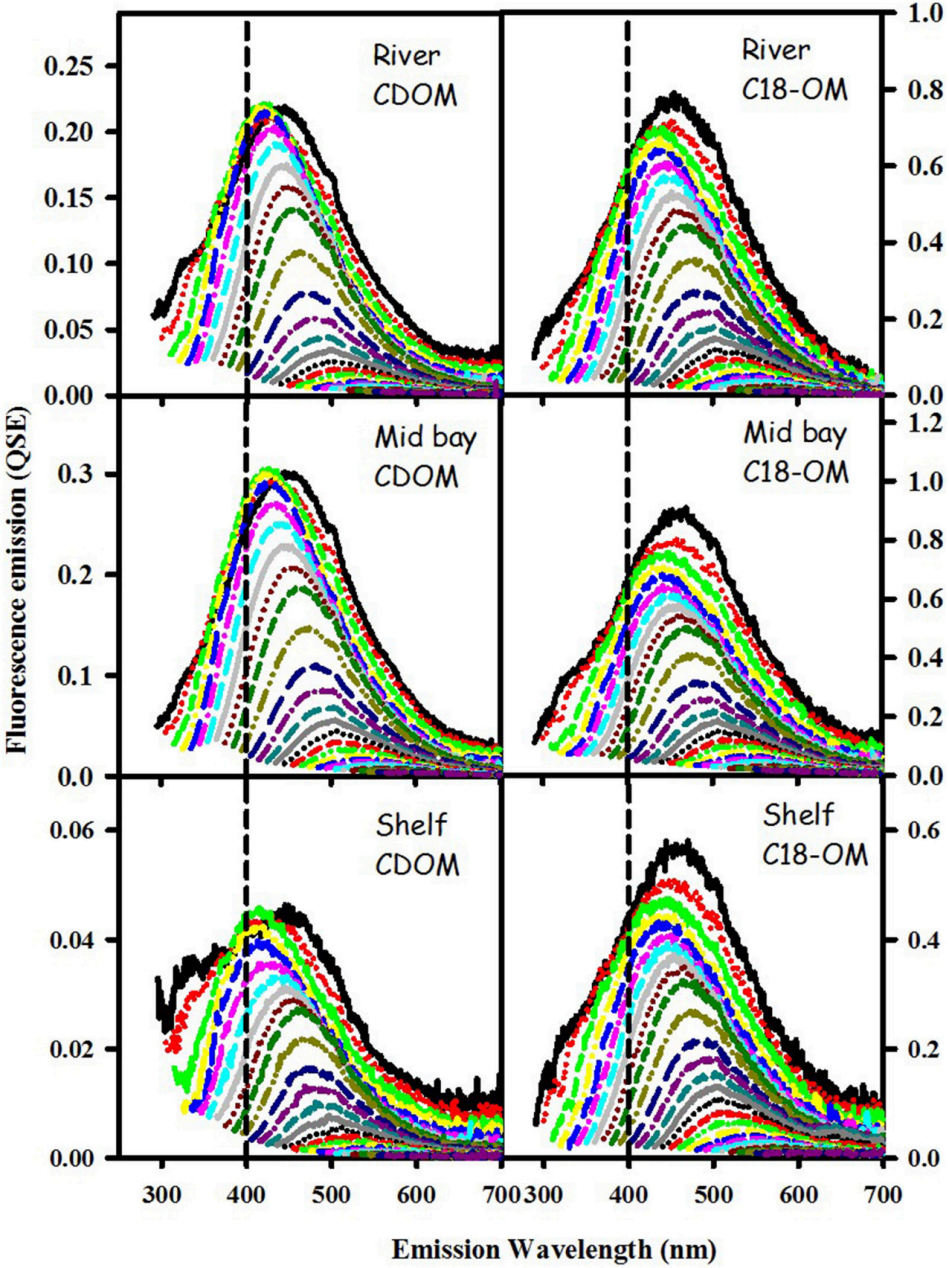
**Corrected fluorescence emission spectra for CDOM and corresponding C18-OM samples from the MAB river, bay, and shelf waters**.

**Figure 4:**
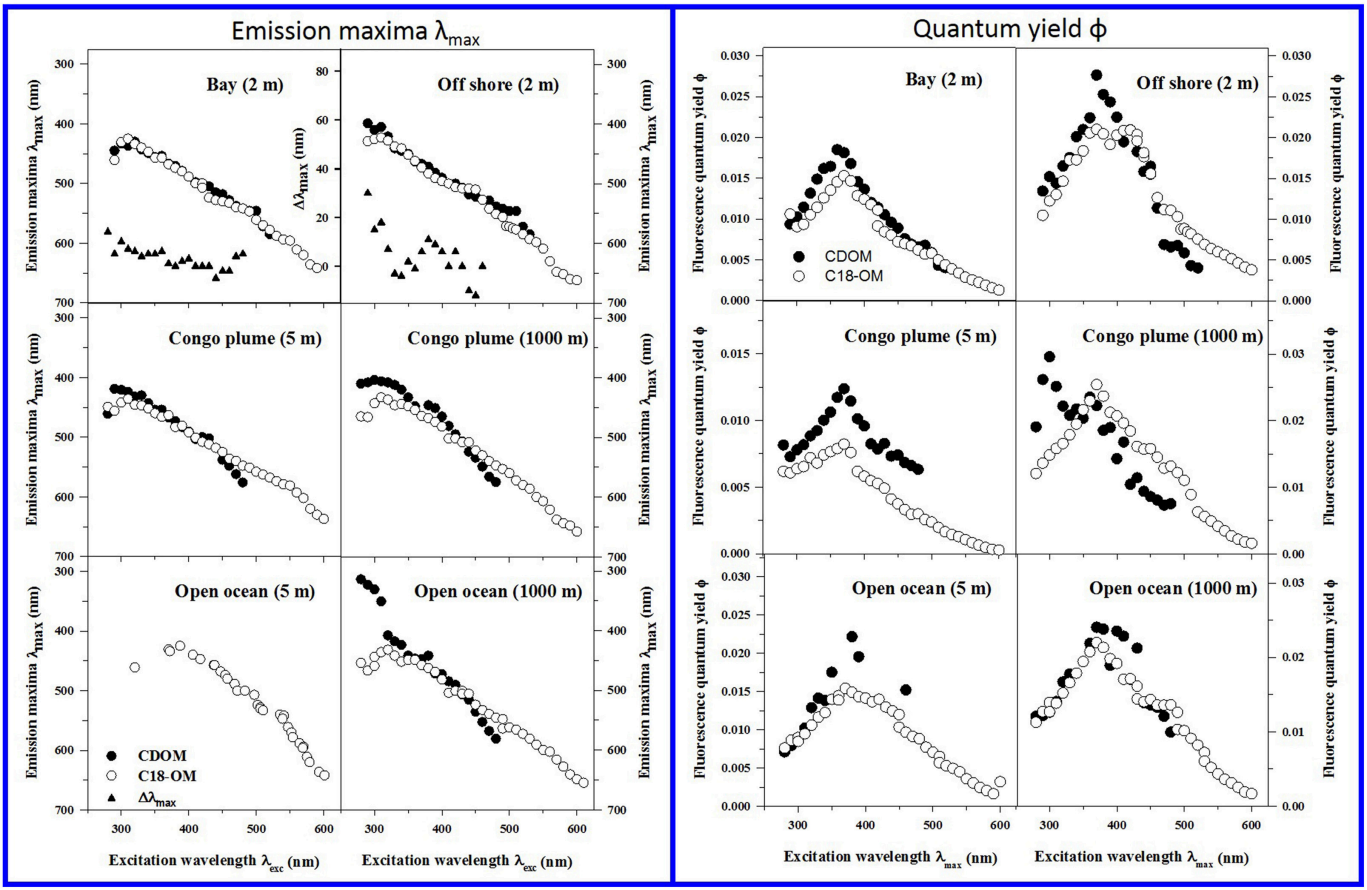
**Corrected fluorescence emission maxima (left panels) and quantum yields (right panels) for representative CDOM (solid symbols) and C18-OM (open symbols) samples from MAB bay and offshore, Congo plume, and open ocean waters**. Emission maxima for CDOM open ocean (5 m) could not be determined due to high signal-to-noise in emission spectra. Emission maxima plots for Bay and Offshore samples also include Δλ_max_ plots; Δλ_max_ > 0, indicates a red shift in λ_max_ for C18-OM relative to CDOM.

CDOM and C18-OM samples from the MAB and EAO exhibited very similar spectral dependencies of fluorescence emission. Emission spectra were broad and unstructured (Figure 3) and their maxima (λ_max_) continuously red-shifted with increasing excitation wavelength (λ_exc_ ≥ ~300 nm) (Figure 4 left panels). Additionally, λ_max_ for C18-OM was generally more red-shifted relative to that of CDOM samples at short wavelengths, suggestive of greater molecular size for C18-OM relative to CDOM, consistent with the absorption measurements.

CDOM and C18-OM exhibited a very similar wavelength dependence of apparent quantum yields (Figure 4 right panels). Values for ϕ increased with increasing excitation wavelength up to ~370 nm, and then decreased monotonically at longer excitation wavelengths. C18-OM samples from the Congo River plume exhibited lower ϕ values at shorter λ_exc_ (<380 nm) relative to CDOM. In contrast, CDOM and C18-OM from the MAB and EAO (open ocean) exhibited comparable ϕ values at λ_exc_ ≤ 370 nm, especially at depth. Effective comparison at longer wavelengths (≥370 nm) could not be made due to very low absorption coefficients in some cases (close to detection limit) or very low fluorescence emission for the CDOM samples resulting in larger uncertainties.

Occasionally discrete peaks were observed in the UV region for samples from the Congo River Plume and from open oceans; these bands exhibited very high quantum efficiencies relative to the bulk CDOM (Figure 4 right panel, Congo Plume 1000 m; Figures 6 and 9 in Andrew et al., 2013), and were not extracted by C18 cartridges indicating that these species are structurally different from the long-wavelength absorbing/emitting CDOM.

### NaBH_4_ reduction

**Figure 5:**
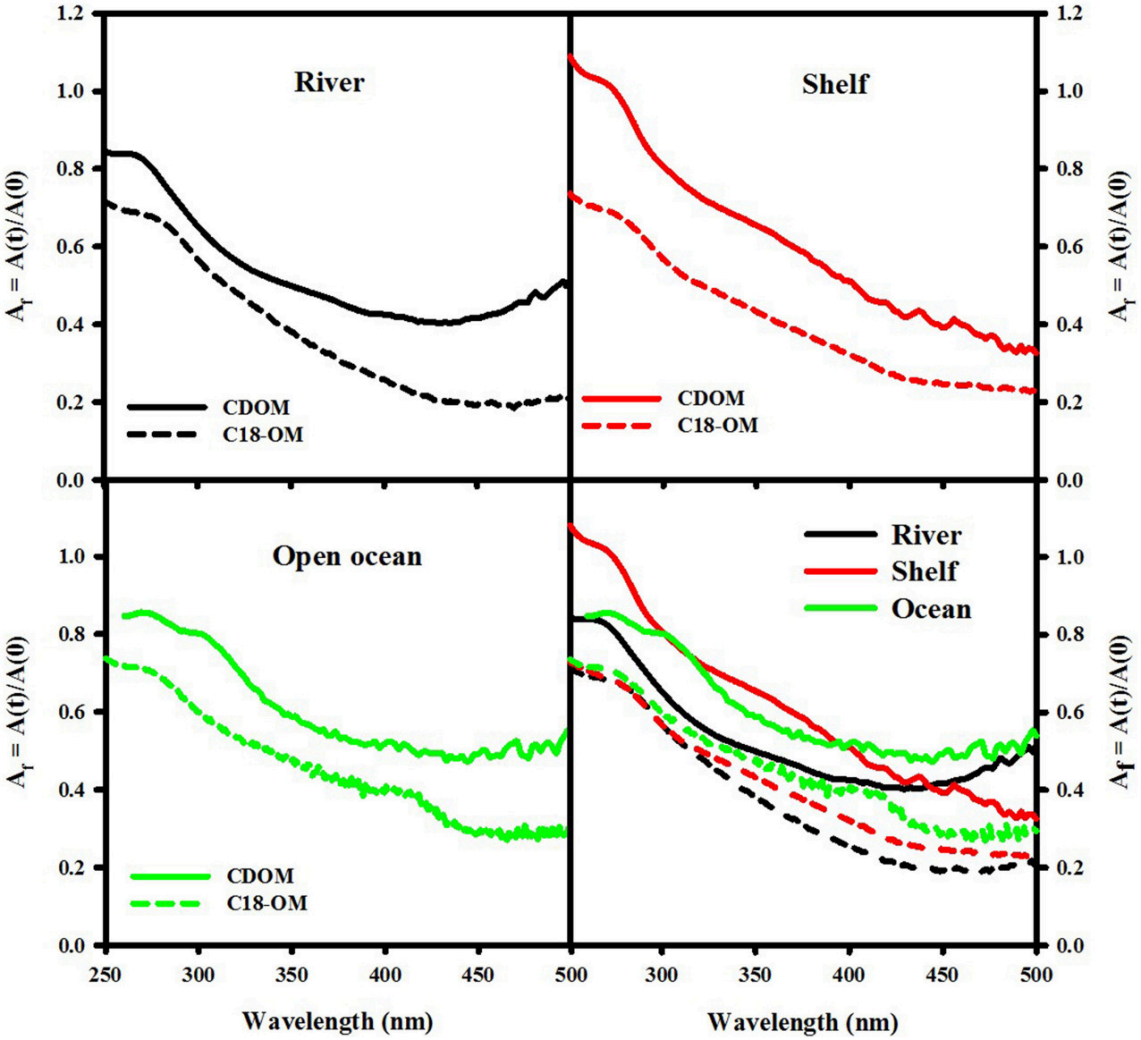
**Wavelength dependence of fractional absorption loss ([A(t)]/[A(0)]) following reduction of CDOM and C18-OM samples from MAB (river—August 2006 St 19, 2 m; shelf—December 2006 St. 9, 20 m) and EAO (June 2009 St 51, 1000 m)**.

Reduction of both CDOM and C18-OM samples with NaBH_4_ resulted in a significant loss in absorbance across the UV-visible wavelengths, with the largest fractional losses observed in the visible wavelength regime (Figure 5) consistent with past observations (Ma et al., 2010; Zhang et al., 2012; Golanoski et al., 2012; Andrew et al., 2013; Baluha et al., 2013; Guo and Ma, 2014; Phillips and Smith, 2014, 2015; Sharpless and Blough, 2014). Absorption losses of ~50% were observed for CDOM from both MAB (river and shelf) and EAO (open ocean). However, much greater losses (as much as 80% over the longer wavelength visible regime) were reported for C18-OM. Although the spectral dependence of fractional absorbance loss looks very similar, CDOM fractional absorbance values were always smaller than the corresponding C18-OM values. This difference may possibly be due to (a) the greater initial content of long-wavelength absorbing material in C18-OM relative to CDOM; or (b) in part be artifactual due to changes in the refractive index (and thus baseline) measured using the long path spectrometer resulting from the addition of borohydride. Thus, the magnitude of fractional loss for CDOM samples may actually be closer to the fractional losses observed for C18-OM.

**Figure 6:**
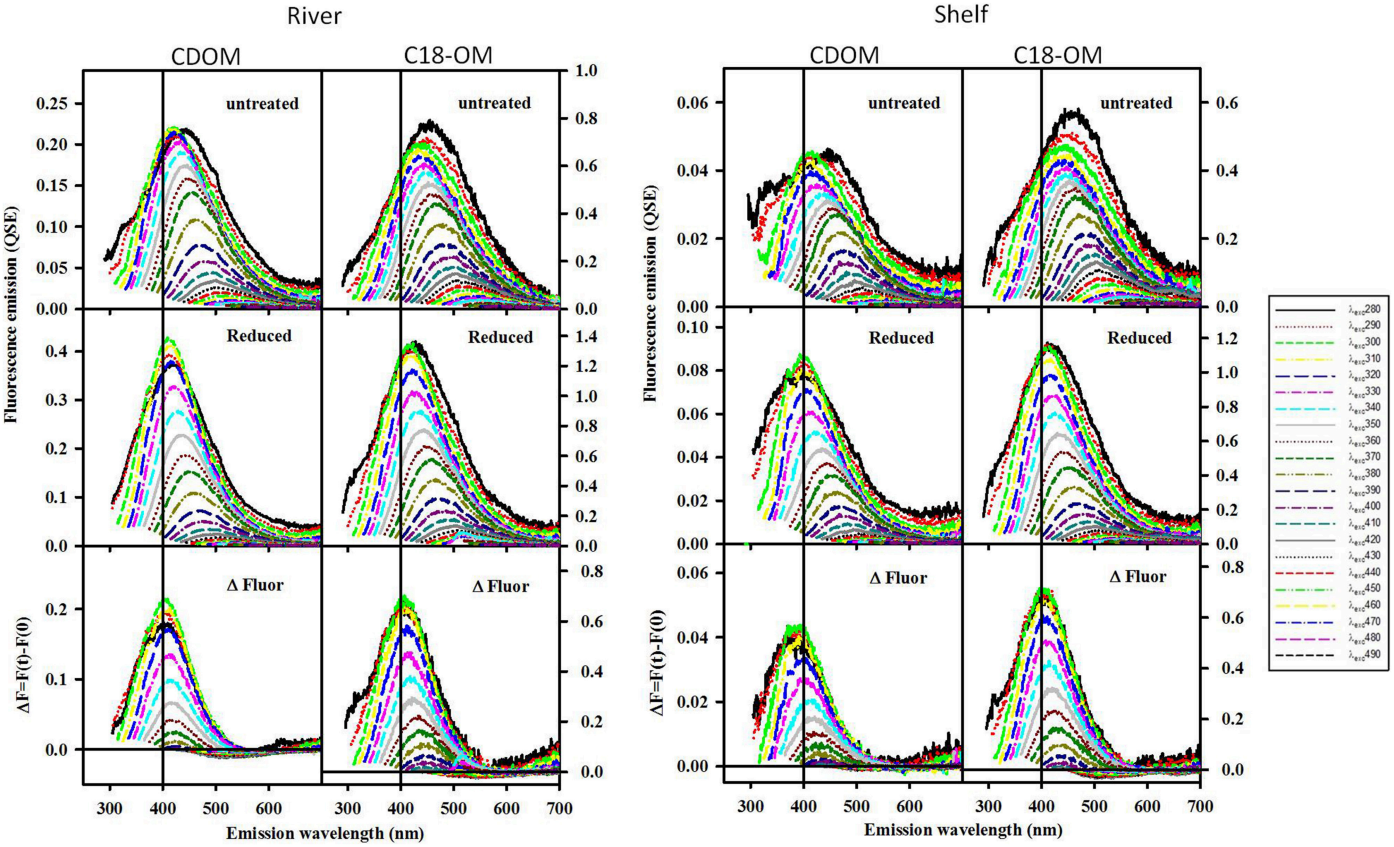
**Corrected fluorescence emission spectra for CDOM and corresponding C18-OM prior to and following reduction with NaBH_4_, for selected MAB samples (river: 40.1 N, 75 W and shelf: 38.4 N, 74.6 W)**. Untreated (Top), Reduced (Middle) and difference (ΔF; Bottom). ΔF > 0 represents increase in emission.

**Figure 7:**
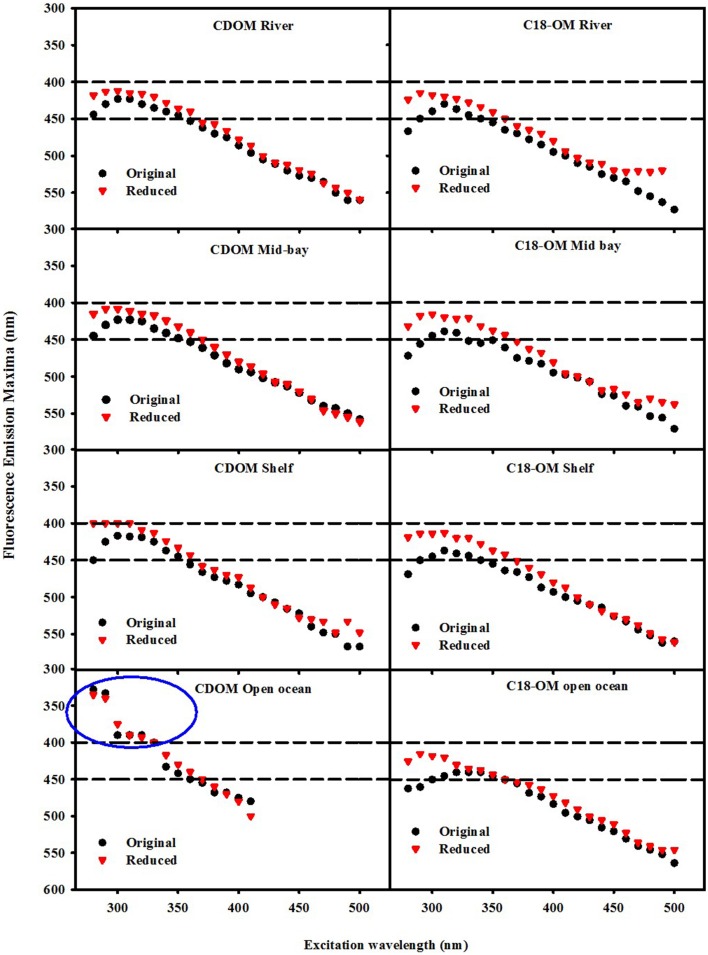
**Wavelength dependence of fluorescence emission maxima λ_max_ for CDOM and C18-OM samples prior to and following NaBH_4_ reduction**. Area in the blue circle represents the emission maxima of a UV emitting species observed in the EAO CDOM sample that failed to be extracted by the C18 cartridges.

**Figure 8:**
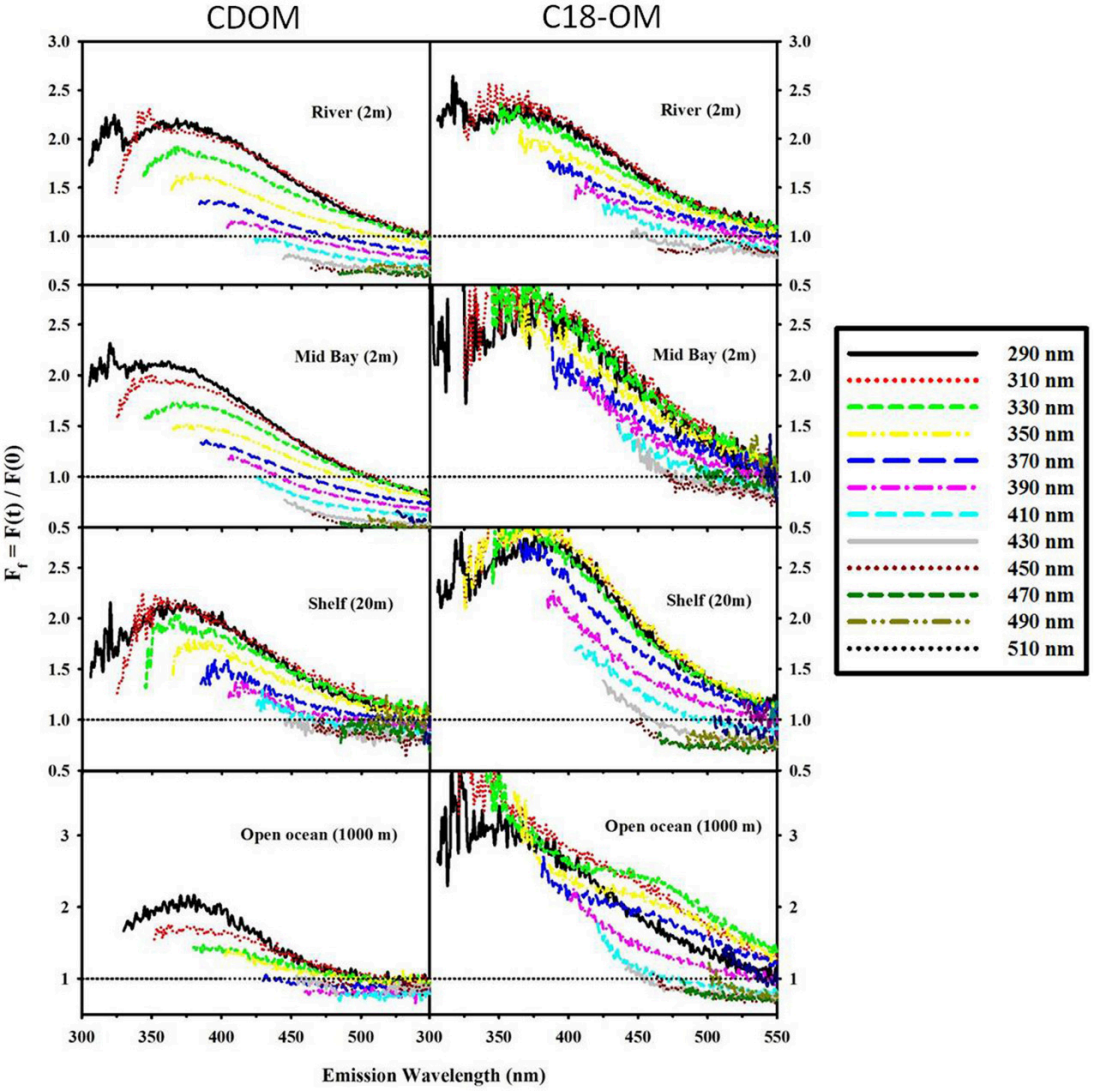
**Fractional fluorescence emission spectra for selected CDOM samples and their corresponding C18-OM: MAB (river: 40.1 N, 75 W; Mid bay: 39.3 N, 75.4 W and shelf: 38.4 N, 74.6 W) and EAO (open ocean: 4.98 S, 0.0 E)**. F_*f*_ > 1 (horizontal black dotted line) signifies an increase in fluorescence after reduction. Spectra are presented for excitation wavelengths every 20 nm starting with λ_exc_ 290 nm.

Following reduction, fluorescence emission intensities increased similarly at short excitation wavelength for both CDOM and C18-OM from the MAB and EAO [MAB Figure 6; EAO Figures 10, 11 in Andrew et al. (2013)] consistent with previous work (Tinnacher and Honeyman, 2007; Ma et al., 2010; Guo and Ma, 2014; Phillips and Smith, 2014, 2015; Sharpless and Blough, 2014). Additionally, the emission maxima shifted to the blue by approximately 10–15 nm relative to the untreated samples (**Figure 7**), with a larger shift observed for C18-OM due to the more red-shifted emission observed in the untreated C18-OM. Loss in emission intensity was observed for both CDOM and C18-OM at longer λ_exc_ (>410 and >430 nm, respectively) (Figure 6 ΔF; **Figure 8** values below the horizontal black line). These results are consistent with results previously reported (Ma et al., 2010; Sharpless, 2012). Overall, the wavelength dependence of fractional changes (gains and losses) in emission intensity before and after reduction were similar for both CDOM and C18-OM samples (**Figure 8**) consistent with the absorption changes upon NaBH_4_ reduction (Figure 5). The greater changes observed for C18-OM samples do not imply different samples' reactivity; instead they could be due to the following factors: (1) Solid NaBH_4_ was added to 30 mL CDOM sample (at low concentration, and neutral pH), thus resulting in kinetic competition between the loss of borohydride through reaction with water and with reduction of carbonyl groups within the CDOM samples, (2) C18-OM is enriched in higher molecular weight material (lower S, more visible absorption at longer wavelengths, greater fluorescence emission for the untreated C18-OM samples) resulting in a greater blue-shift in emission maxima and emission intensity following reduction.

**Figure 9:**
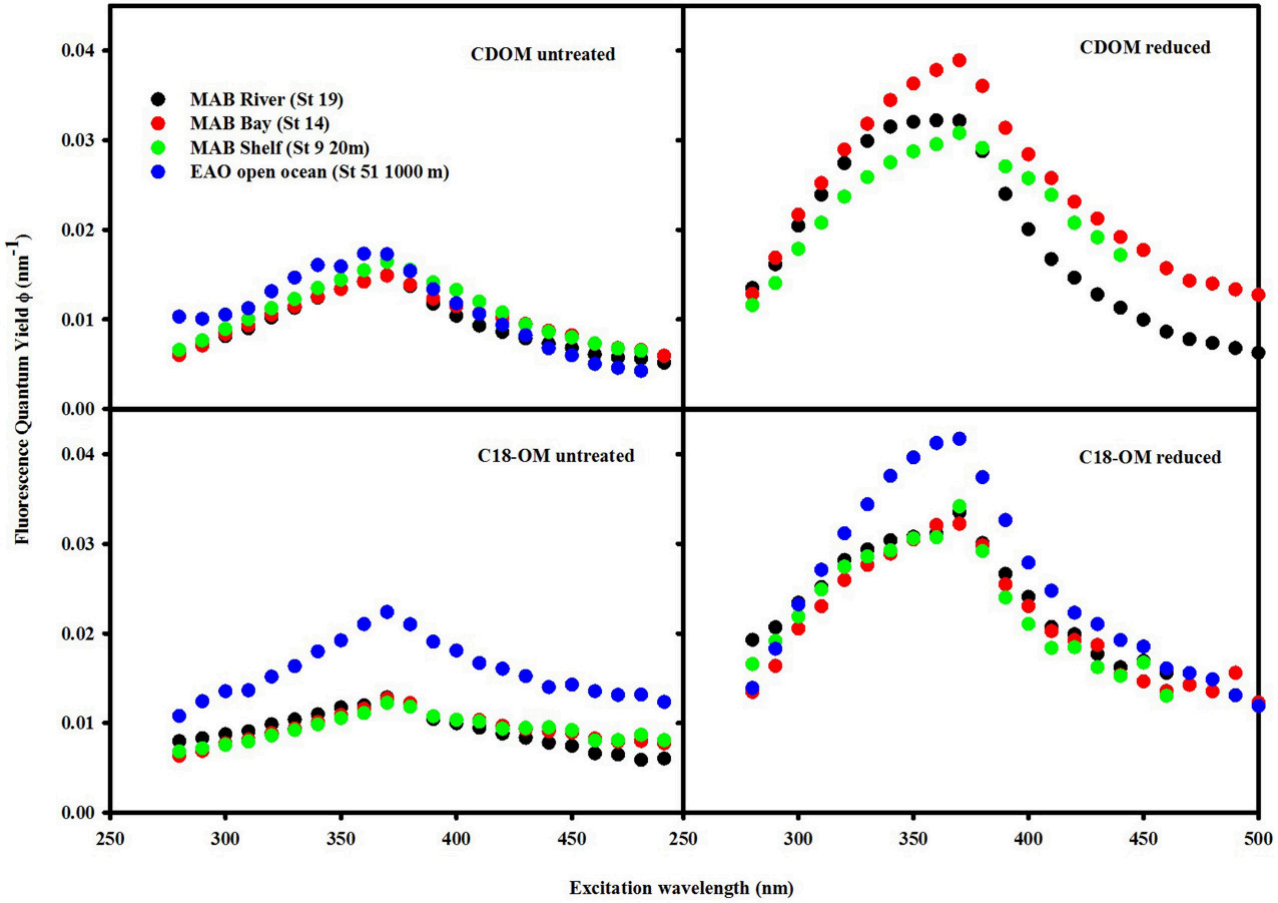
**Wavelength dependence of fluorescence quantum yields for CDOM and C18-OM samples prior to and following NaBH_4_ reduction**. QY values for the reduced CDOM sample EAO (St 51 1000 m) could not be calculated as a result of absorbance values being too low after reduction.

The fluorescence quantum yields for CDOM and C18-OM in the MAB and EAO were substantially enhanced following reduction, particularly at λ_exc_ ~350–390 nm (Figure 9). In previous work by Ma et al. (2010), this enhancement in ϕ was attributed to two factors: (1) the increase in fluorescence emission at λ_exc_ ≤ 350 nm, and (2) the significant decrease in absorption over these same wavelengths, which also holds true for this work as well.

### Comparison of C18-OM to CDOM and comparison among C18-OM

There is slight biasing with the C18 extraction, with some enhancement of absorption in the visible, and some distinct fluorescing components are not extracted. However, the wavelength dependence of absorbance and emission are very comparable, as is the wavelength dependence of the quantum yield. Moreover, C18-OM and CDOM show the similar spectral dependence upon NaBH_4_ reduction, which suggests that these are very similar materials. This indicates that we are not dramatically altering the composition of the CDOM samples upon SPE extraction.

Not only are the C18-OM similar to the original waters, but surprisingly, the C18-OM gathered from very different geographic locales also look very similar, which suggests that there may be a common structural basis amongst all these samples. The wavelength dependence of absorbance, fluorescence (Figure 3, right panel) and emission maxima (Figure 7, right panel) appear to be almost identical. This result suggests that the organic material being extracted is very similar, independent of sample location. The similarity in the changes in optical properties upon NaBH_4_ reduction further highlights this observation. The fractional absorbance spectra (Figure 5), the changes in fluorescence emission maxima (Figures 6, 7) and fractional loss in fluorescence emission (Figure 8) upon reduction are also very similar, indicating a similarity not only in optical properties, but also in chemical behavior and structural origin of the extracted organic matter.

## Summary and conclusion

The extraction efficiency increased with wavelength in areas influenced by fresh water inputs indicative of preferential extraction of longer wavelength absorbing material. Such trend was less evident for offshore waters due to low absorbance values for these samples. Nevertheless, CDOM and C18-OM exhibited remarkably similar wavelength dependence of absorbance, fluorescence emission and fluorescence quantum yields, that appeared to be largely unaffected by the preferential extraction of longer wavelength absorbing material. Thus, these results provide strong evidence that the optical properties of C18-OM and the corresponding CDOM sample are very similar overall.

The results from the NaBH_4_ reduction provide further evidence that the preferential enrichment of long wavelength absorbing material did not largely affect the chemical properties of CDOM and C18-OM. Following reduction, CDOM and C18-OM exhibited comparable changes in fractional absorbance and fluorescence, fluorescence emission maxima and quantum yields that did not vary largely with geographical area.

Remarkably, C18-OM extracts exhibited very similar optical properties and response to chemical reduction independent of location, suggesting a common structural basis for the extracted organic matter.

Overall, the results from this study indicate that enrichment of CDOM by SPE using C18 cartridges does not substantially alter the optical properties of CDOM. Altogether, these results suggest that not only is the structural basis of the optical properties of both the extracted material and original samples comparable, but that similar type of organic material is being extracted independent of sample location. We conclude that C18-OM extracts are representative of CDOM, at least in terms of their optical properties.

## Author contributions

AA, YZ collected, analyzed, and interpreted data. RD, NB, AS contributed to the manuscript writing.

### Conflict of interest statement

The authors declare that the research was conducted in the absence of any commercial or financial relationships that could be construed as a potential conflict of interest.
